# A Facile and Rapid Strategy for Quantifying PCBs in Cereals Based on Dispersive Solid-Phase Extraction and Gas Chromatography–Mass Spectrometry: A Reference for Safety Concerns in Sustainable Textiles

**DOI:** 10.3390/ma16041698

**Published:** 2023-02-17

**Authors:** Tengfei Liu, Ying Song, Xiangyun Wang, Linlin Shi, Minghui Dong

**Affiliations:** 1 Key Laboratory of Detection for Pesticide Residues and Control of Zhejiang Province, Hangzhou 310021, China; 2 Jiangsu Taihu Area Institute of Agricultural Sciences, Suzhou 215105, China

**Keywords:** cereal, polychlorinated biphenyls, carboxylated multi-walled carbon nanotubes, dispersive solid-phase extraction, gas chromatography–mass spectrometry

## Abstract

Cereals and their derivative products such as starch and cyclodextrin are significant natural materials for sustainable textile processing (e.g., sizing, dispersing, etc.). However, the contamination of cereals with polychlorinated biphenyls (PCBs) is often neglected, which has led to increasing concerns due to the adverse effects on end users. Therefore, monitoring PCBs in cereals is of great importance in preventing health risks. However, high starch, protein, and fat contents make cereals a complicated matrix and can challenge the analysis of PCBs in cereals. This work describes a facile and rapid strategy for quantifying 18 PCBs in cereals that included corn, wheat, and rice through dispersive solid-phase extraction and gas chromatography with mass spectrometry. Importantly, this was the first time that carboxyl-modified, multi-walled carbon nanotubes were incorporated in the detection of PCBs in cereals. The influences of several parameters on the extraction and clean-up efficiency were investigated; these included the type and volume of extraction solvent, sonication time, and the type and dosage of the adsorbent. The matrix effects on quantification were also evaluated. This approach exhibited a better clean-up performance. All the analytes showed weak matrix effects, and thus a solvent standard plot could be prepared for their quantification. Spiking experiments in the selected matrices at three concentration levels from 0.5 to 10 μg/kg resulted in satisfactory recoveries that ranged from 79.2% to 110.5% with relative standard deviations (RSDs; *n* = 6) less than 10.3%. The limits of detection (LODs) and quantification (LOQs) ranged from 0.04 to 0.1 μg/kg and 0.1 to 0.4 μg/kg, respectively. The practical application of this method was investigated by analyzing actual cereal samples, which demonstrated that the proposed approach was a facile and efficient strategy for PCB determination and provided a reference for the safety evaluation of sustainable textiles. The method also could be generalized to other troublesome samples for testing of multiple PCBs.

## 1. Introduction

Cereals are key elements of a nutritious and healthy diet [1]. Rice, wheat, and corn are the three staple cereal crops that constitute the main food components of many people’s diets in countries such as China and India [2]. Within the textile domain, cereals and their derivative products are also significant natural materials used to develop the sustainable textile industry. For example, starch, which composes approximately 78% of cereals, is often applied to size cotton yarns prior to weaving. Cyclodextrin derived from cereals is also an important bio-based dispersing agent for dyes or pigments used to replace synthetic surfactants. Many studies have been implemented on the applications of cereals and their derivative products in the treatment of textiles [3,4]; however, their toxicities are often overlooked in most cases. It is widely confirmed that cereals inevitably absorb and accumulate different amounts of contaminants such as heavy metals, pesticide residues, and other chemicals from the environment during their growth. After harvesting, cereals generally go through a process of drying, shelling, milling, etc., which to a certain extent reduces the content of such contaminants, but some of the contaminants still remain in processed grain products [5,6,7]. Numerous studies have documented their existence in cereals on a global scale [8,9,10]. These contaminants can be toxic or even carcinogenic [11,12], and thus their presence in cereals is of public concern. Monitoring these compounds in cereals is thus crucial to protecting humans and animals alike.

Polychlorinated biphenyls (PCBs) are anthropogenic chlorinated chemicals that comprise 209 congeners that differ in the number and position of chlorine atoms on two directly linked biphenyl rings [13]. When added to a material, PCBs offer plastic and fire-retardant properties. They are also very good coolants and lubricating agents. These characteristics have allowed PCBs to be commercially produced for various electrical products such as transformers, capacitors, and voltage regulators since the late 1920s [14]. However, PCBs are highly toxic to humans and may lead to severe health disorders; e.g., neurotoxicity, reproductive and developmental toxicity, and carcinogenic effects [15,16,17,18]. They are also lipophilic and highly persistent [19] and can bioaccumulate in the human body through the food chain [20,21,22] and inhalation [23]. While most countries prohibited the production of PCBs in the late 1970s, PCB legacy materials (e.g., paints, coatings, and plastics) and new materials containing PCBs from pigments with distinct congener pattern continue to be a source of environmental release [24,25,26]. They are still commonly encountered in cereal-based foods [27,28,29,30]. Many countries have established a maximum allowable concentration (MAC) for PCBs in food products. For example, the maximum contaminant level for PCBs in all foods set by the Food and Drug Administration (FDA) is 0.2–3.0 mg/kg [31]. China set the maximum residue limits of 0.5 mg/kg for seven indicator PCBs (28, 52, 101, 118, 138, 153, and 180) in aquatic animals and their products [32]. In view of the large consumption of cereals and the above-described toxicity of PCBs, attention is increasingly being given to the detection and monitoring of PCBs in such samples.

Current analytical methods for the detection of PCBs in cereals primarily rely on the use of gas chromatography with an electron capture detector (GC-ECD) [33,34,35] or GC coupled with mass spectrometry (GC-MS) [27,30,36,37,38,39]. Of these methods, GC-MS is the preferable approach for routine analysis because it is less expensive, easier to operate and maintain, and can offer accurate qualitative and quantitative results for the target analytes. However, sample pretreatment is still required to obtain satisfactory results. This can limit the use of GC-MS with cereal samples because they often contain complex matrices such as lipids and pigments (mainly natural carotenoid and anthocyanidin) [40] that can lead to matrix interferences in GC-MS analysis.

Common sample-preparation procedures for PCB analyses in cereals involve Soxhlet extraction (SOE) [27,30,35,37], accelerated solvent extraction (ASE) [34,38], solid-phase extraction (SPE) [27,34,36,38], dispersive solid-phase extraction (dispersive SPE) [33], and gel permeation chromatography (GPC) [39]. The dispersive SPE method has gained widespread popularity because it is simple and has minimal solvent consumption, operational simplicity, a high throughput, and a low cost. In this procedure, the crude sample extract is directly blended with a small amount of sorbent; the resulting mixture is then vortexed for rapid clean-up effects via interactions between the sorbent and the interfering substances [41]. Thus, the choice and use of the sorbent is crucial. A primary secondary amine (PSA) is the most commonly utilized sorbent. Octadecylsilane (C_18_) and graphitized carbon black (GCB) are also frequently used [42]. Nevertheless, they are usually insufficient for the clean-up of complex matrix samples when applied individually or in combination. This can lead to more maintenance of the instrumentation. These disadvantages prompt the development of new and better sorbents [43]. Multi-walled carbon nanotubes (MWCNTs) have a hollow multi-layered structure, abundant π electrons, and a large surface area [44]. They exhibit a good adsorption ability and are widely employed as dispersive SPE sorbents during food safety analyses of pesticides, veterinary drugs, and metals [45,46,47,48,49]. When functionalized with chemical groups (e.g., carboxyl, hydroxyl, and amine groups), they can gain better characteristics such as a larger surface area, better dispersion, and a greater negative charge on the surface than MWCNTs due to the functional groups [50]. Thus, they have improved application effects. Our previous work demonstrated that MWCNTs functionalized with a carboxyl group (MWCNTs-COOH) showed good clean-up performances and recoveries as a dispersive SPE sorbent for the analysis of PCBs from vegetable and soil samples [51,52] as well as for complex matrices like tea: they could be mixed with other sorbents to increase the clean-up performance of the d-SPE process with impressive results [53]. To the best of our knowledge, however, the use of MWCNTs-COOH either alone or in combination with PSA has not yet been studied as a sorbent for determining PCBs in cereals.

Cereals are high-fat (e.g., linoleic acid) matrices, which makes them difficult to extract and clean. Here, we offer verification studies of a dispersive SPE method for determining PCBs in cereal samples such as rice, wheat, and corn using GC-MS. Eighteen congeners of PCBs (Figure S1) that included indicator PCBs and dioxin-like PCBs were selected for analysis. The parameters that affected the extraction performance, clean-up efficiency, and instrumental analysis were investigated and optimized to achieve reliable and sensitive results. The proposed method was then used to detect PCB residues in real cereal matrices. To our knowledge, this is the first report to describe MWCNTs-COOH and PSA as sorbents to remove interferences from cereals matrices. A novel dispersive SPE method was thus developed for the pretreatment of PCBs residues in cereals, which is unique in the literature.

## 2. Materials and Methods

### 2.1. Chemicals and Reagents

The PCB standard solution of 10 mg/L in n-hexane was obtained from Beijing Tanmo Quality Testing Co, Ltd. (Beijing, China) and included PCBs 28, 52, 101, 118, 153, 138, 180, 81, 77, 123, 114, 105, 126, 167, 156, 157, 169, and 189. The working solutions (0.5–50 μg/L) were prepared via successive dilution of the stock solution with n-hexane and kept at 4 °C. The MWCNTs-COOH (10–30 μm length and 10–20 nm outer diameter) were purchased from Nanjing XFNANO Materials Tech Co., Ltd. (Nanjing, China). The PSA (40–60 μm particle size) was procured from Tianjin Bonna-Agela Technologies Co., Ltd. (Tianjin, China). Solvents including n-hexane, acetonitrile, and acetone were of HPLC grade and were obtained from Tedia (Fairfield, OH, USA). The analytical-grade toluene and anhydrous magnesium sulfate (MgSO_4_) were supplied by Shanghai Sinopharm Chemical Reagent Co., Ltd. (Shanghai, China).

### 2.2. Apparatus

A GC-MS setup equipped with an Agilent 8890 gas chromatograph, a 7693A autosampler, and a 5977B mass detector with an Xtr electron ionization source (Santa Clara, CA, USA) was used for the PCB analysis.

A KQ-500DE ultrasonic cleaner (Kunshan Ultrasonic Corporation, Suzhou, China), a 16R high-speed centrifuge (CenLee Corporation, Changsha, China), an N-EVAP-24 nitrogen evaporator (Organomation Associates, Berlin, MA, USA), and a VM-10 vortex (Daihan Scientific Corporation, Wonju, Republic of Korea) were used to prepare the samples.

### 2.3. Sample Analysis

#### 2.3.1. Sample Pretreatment

The rice, wheat, and corn cereal samples used here were purchased from local markets and grain-planting bases in Suzhou, China. They were ground into flour with a 1000C food grinder (Yongkang RedSun Electromechanical Co., Ltd., Jinhua, China) before analysis. The dry matter content was determined after drying to a constant weight in a drying cabinet at 105 °C; the data are shown in Table S2.

**Scheme 1:**
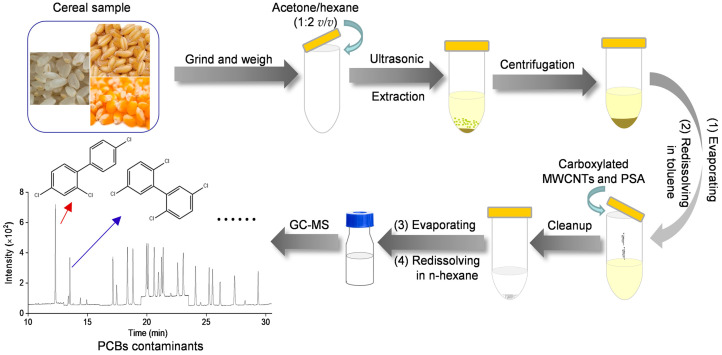
Schematic diagram of the analysis procedure.

First, 2.0 g of the ground sample was weighed into a 50 mL centrifuge tube. After addition of 10 mL of acetone:n-hexane (1:2 *v*/*v*), the sample was then subjected to ultrasonic extraction for 15 min at 500 W using a KQ-500DE ultrasonic cleaner (Kunshan Ultrasonic Corporation, Suzhou, China). After centrifugation at 9000 rpm/min for 3 min, 5 mL of the supernatant was transferred to a 10 mL centrifuge tube and evaporated to nearly dryness using an N-EVAP-24 nitrogen evaporator (Organomation Associates, Berlin, MA, USA) at 80 °C with a flow of 17 mL/min for approximately 8 min. The extract was redissolved in 4 mL of toluene and introduced into a new 10 mL centrifuge tube that contained 0.01 g of MWCNTs-COOH, 0.12 g of PSA, and 0.15 g of anhydrous MgSO_4_. The mixture was then vortexed vigorously for 2 min and centrifuged at 9000 rpm/min for 4 min. All of the supernatant was collected and concentrated to dryness by evaporating it with a weak nitrogen stream at 80 °C, then it was redissolved in 1 mL of n-hexane and filtered through a 0.22 μm nylon membrane for the GC-MS analysis. Quantification was conducted using the external standard calibration method based on direct peak areas alone compared to standards. The detection of PCBs followed the protocol shown in Scheme 1.

#### 2.3.2. GC-MS Analysis

The chromatographic separation used an HP-5MS capillary column (30 m × 0.25 mm × 0.25 µm). Splitless injections (1 µL) were made at an injector temperature of 250 °C. Helium (purity ≥ 99.999%) was used as the carrier gas at a 1.2 mL/min flow rate. The oven temperature program was as follows: begin at 80 °C (hold for 1 min), increase to 180 °C (hold for 2 min) at a rate of 20 °C/min, increase to 230 °C (hold for 2 min) at 3 °C/min, and finally increase to 280 °C (hold for 3 min) at 10 °C/min. The temperatures of the quadrupole, MS interface, and ion source were 150 °C, 280 °C, and 280 °C, respectively. Selected ion monitoring (SIM) mode was applied to the quantitative detection of the target compounds.

### 2.4. Optimization of Extraction and Clean-Up Conditions

To determine the optimal conditions for extracting and purifying the targets from the selected cereals, the following parameters were investigated: (1) extraction solvents: acetonitrile, n-hexane, and mixtures of acetone:n-hexane at 1:1 and 1:2 (*v*/*v*); (2) extraction volumes: 5, 8, 10, and 15 mL; (3) extraction times: 5, 10, 15, and 20 min; (4) toluene volumes: 2, 3, 4, and 5 mL; (5) amount of MWCNTs-COOH: 0.01, 0.02, 0.03, and 0.04 g; and (6) amount of PSA: 0.10, 0.11, 0.12, 0.13, and 0.14 g. Compared to wheat and rice, corn has a more complex matrix (Figure S2). Thus, it was used as the matrix in the optimization experiment and fortified with 18 PCBs at 10 μg/kg. The recoveries of the targets were selected as the evaluation indices.

### 2.5. Evaluation of the Matrix Effect

The matrix effect (ME) for the different cereal matrices (corn, wheat, and rice) was evaluated by comparing the ratios of the slope of the matrix-matched calibration curve with that of the n-hexane calibration curve according to following equation:

(1) $$ME(\%)=\left(\frac{S_{m}}{S_{s}}-1\right)\times 100$$

where *S_m_* and *S_s_* are the slopes of the calibration curves in the matrix and n-hexane, respectively.

### 2.6. Method Validation

The method was validated according to SANTE/11312/2021 guidance [54], which involved the investigation of the specificity, linearity, accuracy, precision, and limits of detection (LODs) and quantification (LOQs). The specificity of the method was evaluated by analyzing the blank sample extracts in which the target analytes should have been differentiated from the interferences. The linearity of the method was evaluated by obtaining calibration plots of the targets with six calibration standards prepared in n-hexane (0.5, 1, 5, 10, 20, and 50 μg/L). A recovery experiment was used to determine the accuracy and precision of the method. There were six replicate analyses for each matrix at spiked concentrations of 0.5, 5, and 10 μg/kg. The accuracy was estimated according to the recoveries (%), and the precision was evaluated according to the relative standard deviations (RSDs, %) of the spiked samples. The LODs were determined as the concentration of analyte that resulted in a signal-to-noise ratio (S/N) of 3:1. The LOQs were defined as the concentration of analyte that produced an S/N of 10:1 [55].

### 2.7. Statistical Analysis

The data in this study were expressed as the means ± standard deviation and subjected to statistical analysis using SPSS version 19.0 (SPSS Inc., Chicago, IL, USA). The main effects were analyzed and the means were compared using Duncan’s multiple range tests at a significance level of 0.05.

## 3. Results and Discussion

### 3.1. Optimization of GC-MS

**Figure 1:**
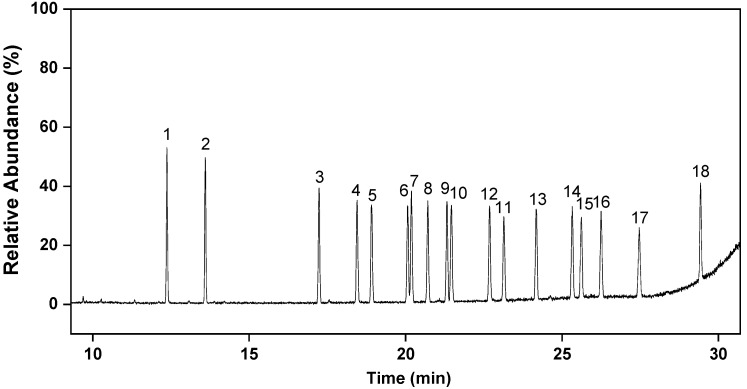
Total ion chromatogram of the 18 PCB standard solutions at 500 μg/L. Peak identification: (1) PCB 28; (2) PCB 52; (3) PCB 101; (4) PCB 81; (5) PCB 77; (6) PCB 123; (7) PCB 118; (8) PCB 114; (9) PCB 153; (10) PCB 105; (11) PCB 138; (12) PCB 126; (13) PCB 167; (14) PCB 156; (15) PCB 157; (16) PCB 180; (17) PCB 169; (18) PCB 189.

The 18 PCBs were selected based on the WHO International Agency for Research on Cancer and GEMS/Food Programme recommendations. There were 12 dioxin-like PCBs and 6 indicative PCBs, which were a group of nonpolar substances with benzene rings [56]. To select the appropriate separation conditions, a series of experiments was performed with working standard solutions that each contained a PCB congener at 500 μg/L. Satisfactory separation of the analytes was obtained using a HP-5MS capillary column with an optimal programmed temperature, inlet temperature, and carrier gas flow rate (see details in Section 2.3.2). The chromatogram of the target compounds is shown in Figure 1.

**Figure 2:**
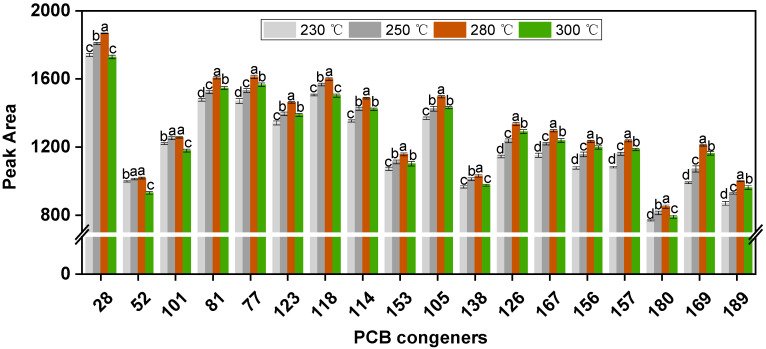
Effect of ion source temperature on the response intensity of the target PCBs. Different lowercase letters indicate significant differences at *p* < 0.05 under different treatments.

Optimization of the MS conditions was important to acquiring the maximum sensitivity when identifying and quantifying the target compounds. Thus, the signal strengths of the analytes and their stabilities at different ion source temperatures were investigated. Figure 2 shows that the optimum temperature of the ion source was determined to be 280 °C. The total ion chromatogram (TIC) was achieved by analyzing 500 μg/L of the working standard solution at a *m*/*z* range of 150–500 via GC-MS in full-scan mode. TIC was used to study the retention time of each analyte. This was identified by searching the NIST mass spectrum library. To obtain the maximum response for each PCB congener, the analytes were divided into many groups based on their retention times in the MS acquisition method. One quantitative ion and two qualitative ions were selected to determine each PCB congener. A good peak shape and a higher detection sensitivity were obtained under these conditions. The selected quantitative ion and qualitative ions for each of the 18 PCBs are presented in Table S1.

### 3.2. Optimization of Extraction Procedure

**Figure 3:**
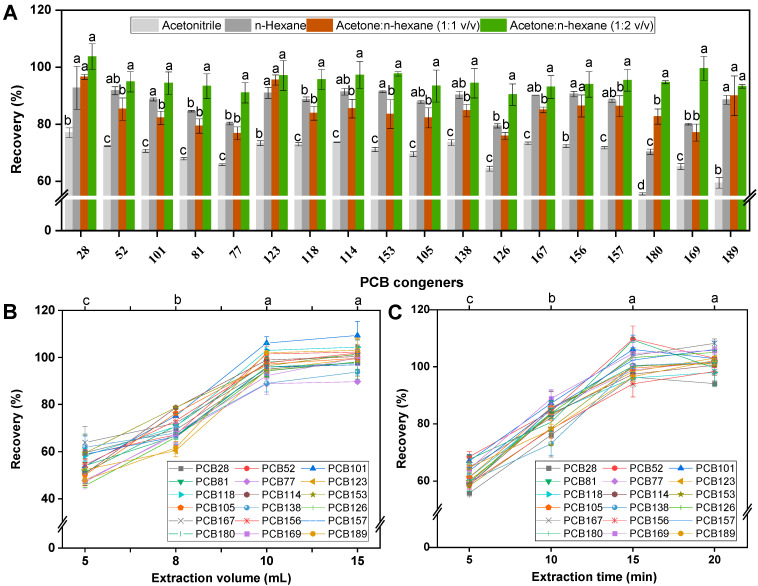
Effects of (**A**) type of extraction solvent, (**B**) volume of extraction solvent, and (**C**) extraction time on extraction efficiency (*n* = 3). Different lowercase letters indicate significant differences at *p* < 0.05 under different treatments.

Selecting a suitable extraction solvent can improve the extraction efficiency of analytes. In this study, the impact of several organic solvents on the extraction efficiency were investigated; these included acetonitrile, n-hexane, and mixtures of acetone:n-hexane at 1:1 and 1:2 (*v*/*v*). These were most frequently used in previous analyses of PCBs from different matrices [37,57,58,59,60,61,62]. It can be seen in Figure 3A that the recoveries of the 18 PCBs were poor when acetonitrile was used as the extraction solvent. The recoveries of PCBs 180 and 189 were only 55% and 59%, respectively. However, the extraction efficiencies were much better when using the other extraction solvents. The recoveries of the target analytes ranged from 70% to 92% for n-hexane, from 75% to 96% for acetone:n-hexane (1:1 *v*/*v*), and from 91% to 103% for acetone:n-hexane (1:2 *v*/*v*). The extraction efficiencies when using acetone:n-hexane (1:2 *v*/*v*) were clearly the best; this may be attributed to this mixture’s better solvation capability with the target compounds and penetrability into the pores of the sample matrix. Thus, this mixture was adopted as the extraction solvent for subsequent experiments.

The volume of extraction solvent can also affect the extraction efficiency. To select the appropriate volume of the extraction solvent, different volumes of acetone:n-hexane (1:2 *v*/*v*) were tested. The data in Figure 3B show that the extraction efficiency of the target analytes increased as the volume of acetone:n-hexane (1:2 *v*/*v*) was increased. When 10 mL of acetone:n-hexane (1:2 *v*/*v*) was used, recoveries of each of the analytes were satisfactory and within the acceptable range (88–106%). The recovery rates did not show marked changes with further increases in the volume of the extraction solvent (*p* > 0.05). Therefore, 10 mL of acetone:n-hexane (1:2 *v*/*v*) was utilized in further experiments to obtain an economic and effective extraction procedure.

Ultrasonic extraction (UE) is commonly used as an extraction technique because it is simple, affordable, and can pretreat many samples concurrently with no generation of chemical waste. To extract the greatest number of analytes, the effect of sonication time on the recoveries of the targets was examined from 5 to 20 min. Figure 3C shows that the recoveries increased as the sonication time was increased from 5 to 15 min. There were no further significant enhancements (*p* > 0.05) of the extraction efficiency when the sonication time was extended beyond 15 min. This might be because the kinetics of UE when extracting these PCBs were very fast and reached equilibrium at 15 min. Indeed, the recoveries of some of the target analytes (PCBs 28, 52, 101, and 180) even decreased. This phenomenon could be due to the longer time of disrupting the equilibrium. With 15 min of sonication time, the recoveries of the target analytes were within the acceptable range of 94–109%. Thus, 15 min was chosen as the extraction time in the following experiments.

### 3.3. Optimization of the Clean-Up Procedure

**Figure 4:**
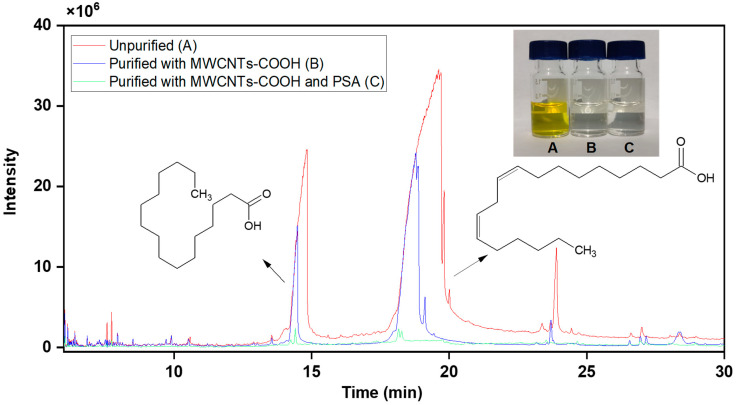
Comparison of GC-MS total ion chromatograms of corn extracts purified with different adsorbents. (**A**) unpurified, (**B**) purified with MWCNTs-COOH, and (**C**) purified with MWCNTs-COOH and PSA.

Cereal crops have complex compositions such as high starch, protein, and fat contents [63]. In particular, corn has a high content of pigments that may be co-extracted with the analytes, thus resulting in severe matrix interference and instrument contamination that may hinder analyte identification and confirmation. The extracts from such samples thus need to be cleaned to eliminate these co-extractives prior to analysis. Here, a dispersive SPE method using specific sorbents was applied for clean-up. The new carbon-based MWCNTs-COOH nanomaterial has been used in many studies when applying the dispersive SPE procedure because it can provide prominent clean-up performance [51,52]. This material has a hollow cylindrical structure and hence a large surface area, thus enabling it to efficiently adsorb matrix impurities (especially pigments and fatty acids) [64]. This adsorption occurs via two types of interactions: adsorption on the surface and the absorptive action of the nanotubes [55]. However, the application of MWCNTs-COOH to dispersive SPE sample pretreatment has rarely been reported for the clean-up of crude extracts for the analysis of PCBs in cereals and was therefore worthy of investigation. Here, the purification effect of MWCNTs-COOH on corn extract was evaluated via color removal and chromatographic analysis. The results, which are shown in Figure 4A,B, exhibited that the crude corn extract was turbid and yellow. However, the cleaned extract appeared to be almost colorless and transparent when pretreated with MWCNTs-COOH, thus implying a good clean-up performance of the sample matrix in terms of the removal of impurities such as pigments. Figure 4 also shows a TIC of the corn extract after clean-up with MWCNTs-COOH. After purification with MWCNTs-COOH, two large interfering peaks persisted in the chromatogram of the blank corn extract and severely affected the accuracy. These interferences, which were mainly due to fatty acids (linoleic acid and palmitic acid), were identified after spectral analysis via comparison with standard mass spectra from the NIST mass spectrum library. Fatty acids in the extract obtained from the corn sample were not removed by the MWCNTs-COOH; thus, it was necessary to evaluate the impact of the PSA sorbent addition during clean-up. PSA is a weak anion exchanger that is frequently used in dispersive SPE methods to adsorb fatty acids, sugars, and other components from the sample matrix. Therefore, PSA was mixed with MWCNTs-COOH to increase the removal of interferences from the corn extract. Two large interference peaks disappeared from the chromatogram after purification with MWCNTs-COOH and PSA (Figure 4C). The baseline became lower than that for samples with MWCNTs-COOH clean-up alone, thus indicating that the PSA had a better adsorption efficiency for fatty acids in the sample matrix. The experiment was consequently designed to utilize the combination of MWCNTs-COOH and PSA as sorbents to purify the cereal extracts.

The amounts of MWCNTs-COOH and PSA were optimized to obtain high recoveries and good purification effects. Our preliminary experiment showed that MWCNTs-COOH could lead to the greatest loss in PCBs (especially for PCBs 81, 77, 126, and 169). This in turn greatly reduced the recoveries of the target analytes because PCBs are a family of compounds that possess benzene ring structures. These analytes were then adsorbed by MWCNTs-COOH through van der Waals forces and/or π–π stacking effects. To solve this problem, the clean-up procedure was conducted with toluene as the extraction solvent in place of acetone:n-hexane (1:2 *v*/*v*). The benzene ring of toluene could compete with the nanotubes and reduce the tendency of PCBs to become adsorbed on the latter. However, the excessive use of toluene may lead to a decline in the recovery of the target compounds. In this study, the recovery effect of toluene at 2, 3, 4, and 5 mL used to redissolve the corn extracts spiked with PCBs was evaluated.

**Figure 5:**
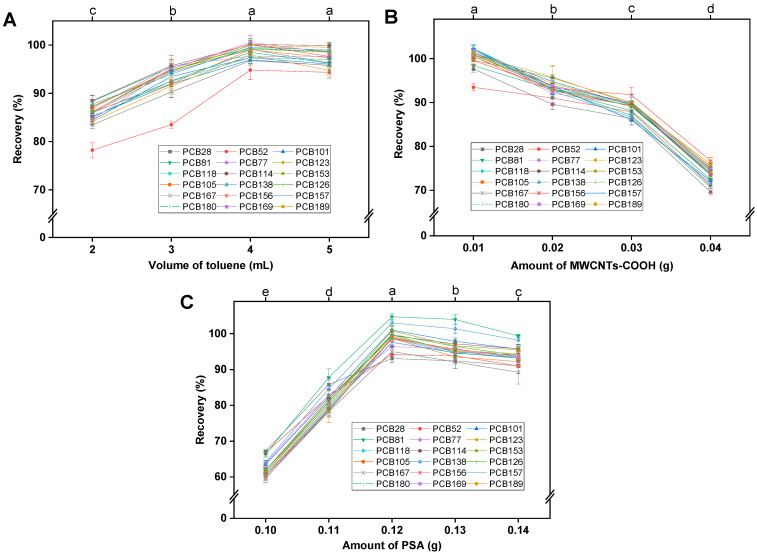
Effects of (**A**) volume of toluene, (**B**) amount of MWCNTs-COOH, and (**C**) amount of PSA on purification performance (*n* = 3). Different lowercase letters indicate significant differences at *p* < 0.05 under different treatments.

As shown in Figure 5A, the recoveries of the target analytes increased when the volume of toluene ranged from 2 to 4 mL and were almost stable or slightly decreased when it was further raised to 5 mL. Thus, a volume of 4 mL was chosen as the optimal volume for toluene. Recovery experiments were performed next on aliquots (5.0 mL) of the corn extract with toluene solvent and purified with different amounts of MWCNTs-COOH and PSA, respectively. The goal here was to select an appropriate sorbent amount. The results in Figure 5B show that the recoveries of all analytes exhibited a downward trend as the amount of MWCNTs-COOH was increased, which indicated that this material adsorbed PCBs while removing matrix impurities. In the case of 0.01 g of MWCNTs-COOH, the final corn extracts were almost transparent in color (Figure 4B), and the best recoveries (between 93% and 102%) were obtained. Meanwhile, the recoveries of PCBs showed a firstly increased then decreased trend with an increment in the amount of PSA (Figure 5C). This might be because the separation procedure became difficult with the increase in the PSA amount. When 0.12 g of PSA was used, less interference was observed in the chromatogram of the corn extract, and the recoveries were at an acceptable level (93–105%). Therefore, 0.01 g of MWCNTs-COOH and 0.12 g of PSA were recommended as the optimal amounts in the clean-up procedure.

Based on the aforementioned discussion, the optimum conditions for the extraction and clean-up procedures were as follows: 10 mL acetone:n-hexane (1:2 *v*/*v*) as the extraction solvent and ultrasonic extraction for 15 min; 4 mL toluene as the solvent for redissolving the extract; and 0.01 g MWNCTs-COOH and 0.12 g PSA as the dispersive SPE sorbents. All of the following experiments were performed under these conditions.

### 3.4. Matrix Effect

**Figure 6:**
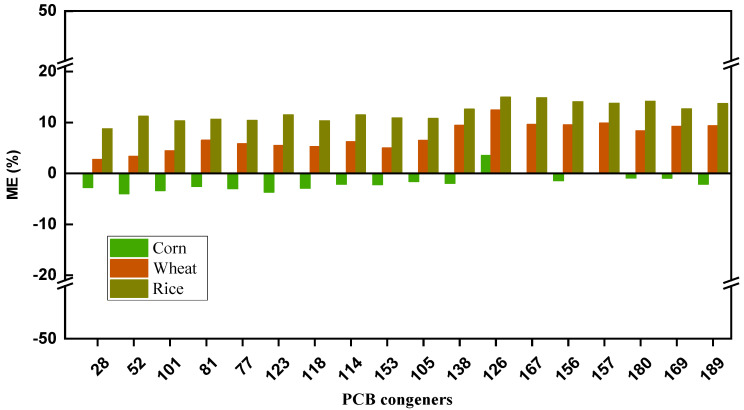
The matrix effects of 18 PCBs in corn, wheat, and rice.

It is essential to investigate the matrix effect (ME) because the matrix can significantly interfere with the analytical process and affect the accuracy of analytical results. Values of the ME between −20% and 20% represent soft matrix effects, which are negligible; values between −50% and −20% or 20% and 50% represent medium matrix effects; and values less than −50% or higher than 50% represent strong matrix effects [65]. As demonstrated in Figure 6, the values of the ME for the PCBs in corn, wheat, and rice were −0.076% to −4.01%, 2.73% to 12.41%, and 8.76% to 14.85%, respectively. Thus, all of the analytes showed a negligible ME, which indicated that the pretreatment method could efficiently eliminate the matrix interferences. This means that the proposed method can be applied to the quantification of PCBs in corn, wheat, and rice matrices using a solvent standard plot.

### 3.5. Validation of the Method

#### 3.5.1. Specificity

**Figure 7:**
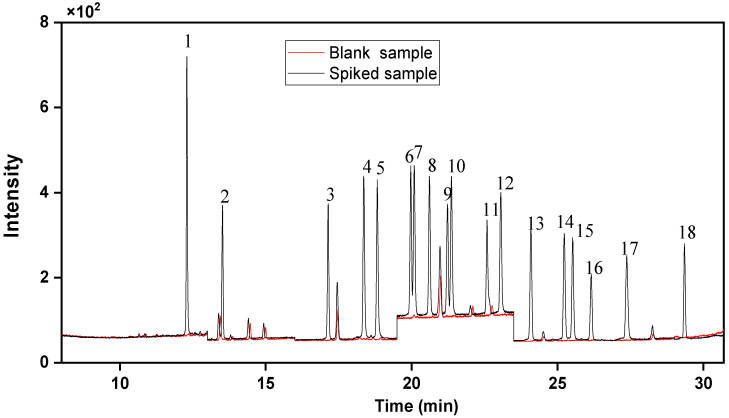
SIM chromatograms of a blank corn sample (red) and a spiked corn sample at 10 μg/kg. Peak identification: (1) PCB 28; (2) PCB 52; (3) PCB 101; (4) PCB 81; (5) PCB 77; (6) PCB 123; (7) PCB 118; (8) PCB 114; (9) PCB 153; (10) PCB 105; (11) PCB 138; (12) PCB 126; (13) PCB 167; (14) PCB 156; (15) PCB 157; (16) PCB 180; (17) PCB 169; (18) PCB 189.

A representative chromatogram of the PCBs extracted from corn is shown in Figure 7 (sample spiked at 10 μg/kg for each PCB congener). No interfering peaks were observed at the same retention times as the analytes. All of the analytes could be differentiated from the other compounds present in the sample, thus confirming that the method was selective.

#### 3.5.2. Linearity

As presented in Table S2, the linearities of the 18 PCBs were desirable and had correlation coefficients (*r*) that ranged from 0.9978 to 0.9986, which indicated a strong correlation between the peak area and the concentration of each analyte.

#### 3.5.3. LODs and LOQs

As illustrated in Table S2, the LODs and LOQs for the targets in the selected matrices ranged from 0.04 to 0.1 μg/kg and 0.1 to 0.4 μg/kg, respectively, which suggested a good sensitivity of the method.

#### 3.5.4. Accuracy and Precision

The mean recoveries shown in Table S3 at different spiking levels varied from 79.2% to 110.5%. An acceptable precision was also achieved with RSDs ranging from 1.5% to 10.3%. Thus, this method can be considered accurate and efficient with good repeatability for the detection of PCBs in cereal samples.

### 3.6. Method Performance Comparison

The parameters of the developed method were compared with other reported methods for the analysis of PCBs in cereal samples in terms of applicability for the simultaneous determination of multiple targets, matrix examined, pretreatment method and time, solvent consumption, LODs, and method of detection. The results in Table 1 show that this novel protocol is convenient, fast, and low-cost with a simpler pretreatment procedure, shorter preparation time, and lower consumption of organic solvents than most of the listed methods. Moreover, this method does not require the use of bulky or expensive equipment such as SOE, ASE, or GPC, which greatly reduces the cost of analysis. It can be conducted in an ordinary testing laboratory. Although this method consumes more organic solvents than the method found in the literature [33], it has significant advantages in determining multiple PCBs and eliminating false qualitative detection of the analytes, thus improving its accuracy.

### 3.7. Method Application

The method was used to analyze real cereal samples. Sixteen corn samples, fifteen wheat samples, and fifteen rice samples were collected from local markets and grain-planting bases in Suzhou, China. These samples were treated using the method described in Section 2.3.1 and analyzed via GC-MS. Two of the PCB congeners (PCB 118 and PCB 138) were detected in one corn sample at 8.9 and 5.5 μg/kg, respectively. The detected concentrations were much lower than the MAC values set by the FDA for PCBs in food [31]. The concentrations of the other target PCB congeners in these cereal samples were all below the LODs.

## 4. Conclusions

Cereals present complex samples that require difficult extraction and clean-up methods during PCB analysis. Here, a very simple and reliable dispersive SPE method combined with GC-MS was used to determine 18 PCBs in corn, wheat, and rice. A mixture of MWCNTs-COOH and PSA was employed in the clean-up step to obtain a better clean-up performance. The results showed that MWCNTs-COOH could increase the removal of interfering substances—especially pigments—from the cereal extracts. Toluene was added in the dispersive SPE process to improve the recoveries of the analytes. Compared to the methods used in other studies that involved PCB analysis in cereal crops, this method has value in terms of the target number, sample preparation procedure, and method validation. Indeed, a satisfactory linearity, accuracy, precision, LODs, LOQs, and matrix effect were all obtained, thus demonstrating the suitability of this method for multi-residue PCB analysis in cereals, which provides a reference for the safety evaluation of sustainable textiles that also could be generalized to other troublesome samples for the testing of multiple PCBs.

## Figures and Tables

**Table 1:** Comparison of the developed method with previously reported methods.

Matrix	Number of PCB Congeners	Pretreatment Method	Pretreatment Time (min)	Solvent Consumption (mL)	Detection Method	LODs (μg/kg)	Reference
Rice and wheat	33	SOE and alumina/silica SPE	>1440	>50	GC–MS	0.03–0.1	[27]
Rice, wheat, and corn	7	UE and dispersive SPE (PSA + C18E + GCB)	<30	5	GC-ECD	-	[33]
Rice, wheat, corn, soybean, and black bean	8	ASE and GPC/Florisil SPE	>45	>20	GC-ECD	0.07–0.3	[34]
Rye and wheat	15	SOE and oleum (7% SO_3_ in concentrated H_2_SO_4_)	>480	120	GC-MS	-	[37]
Rice, wheat, and corn	18	UE and dispersive SPE (MWCNTs-COOH + PSA)	≈37	15	GC-MS	0.04–0.1	This work

## Data Availability

Not applicable.

## Supplementary Materials

The following supporting information can be downloaded at: https://www.mdpi.com/article/10.3390/ma16041698/s1, Figure S1: Structural formula of the selected PCB congeners; Figure S2: Total ion chromatogram of corn, wheat, and rice extracts; Table S1: Retention time, qualitative ion, and quantitative ion of target PCBs; Table S2: Linear ranges, regression equations, correlation coefficients, LODs, and LOQs of 18 PCBs; Table S3: The recoveries and RSDs of the proposed method for the spiked samples at different levels.
