# Supplementary material for: A Facile and Rapid Strategy for Quantifying PCBs in Cereals Based on Dispersive Solid-Phase Extraction and Gas Chromatography–Mass Spectrometry: A Reference for Safety Concerns in Sustainable Textiles
Source: Materials (Basel). 2023 Feb 17;16(4):1698. doi: 10.3390/ma16041698 (PMC9961118; doi:10.3390/ma16041698)
Supplement: Supplementary file 1 [file materials-16-01698-s001.zip › materials-2202680-supplementary.pdf]

A Facile and Rapid Strategy for Quantifying PCBs in Cereals Based on Dispersive Solid-Phase Extraction and Gas Chromatography-Mass Spectrometry: A Reference for Safety Concern in Sustainable Textiles

Tengfei Liu [1,2] , Ying Song [2] , Xiangyun Wang [1,*], Linlin Shi [2], Minghui Dong [2]

[1] Key Laboratory of Detection for Pesticide Residues and Control of Zhejiang Province, Hangzhou 310021, China; liutengfei@jaas.ac.cn (T.L.)
[2] Jiangsu Taihu Area Institute of Agricultural Sciences, Suzhou 215105, China; songyingkjc@sina.com (Y.S.); 20113002@jaas.ac.cn (L.S.); dmhsaas@163.com (M.D.)
* Correspondence: wxy_zaas@163.com (X.W.)

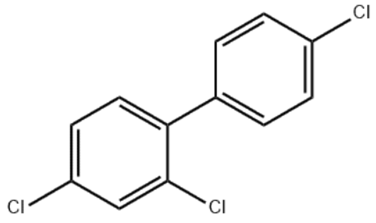

PCB 28

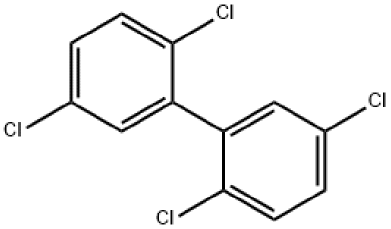

PCB 52

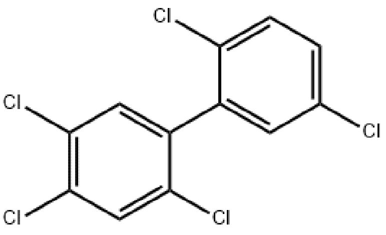

PCB 101

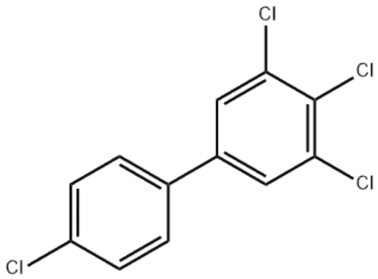

PCB 81

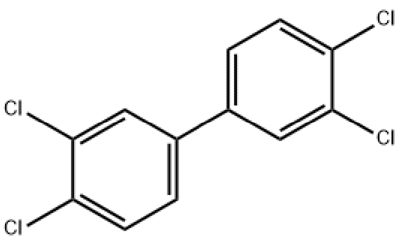

PCB 77

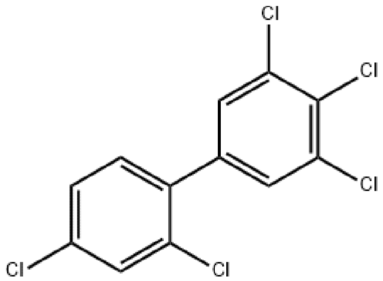

PCB 123

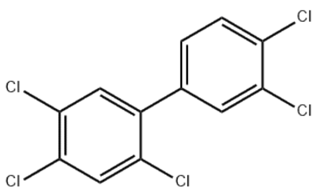

PCB 118

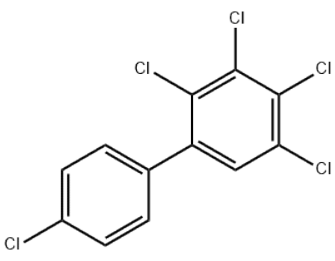

PCB114

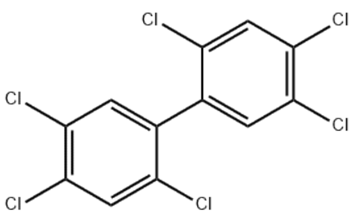

PCB 153

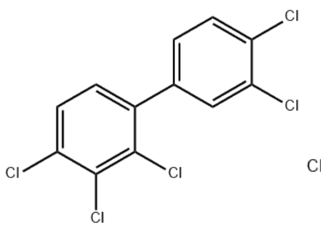

PCB 105

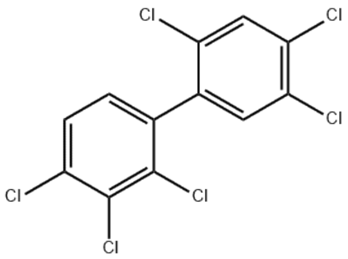

PCB 138

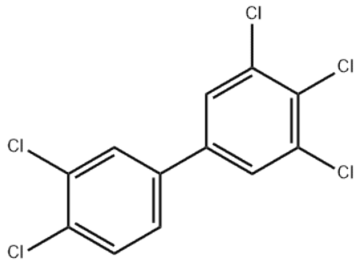

PCB 126

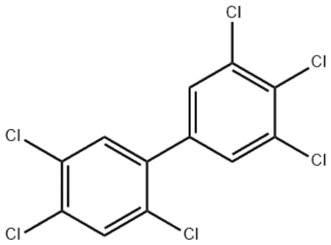

PCB 167

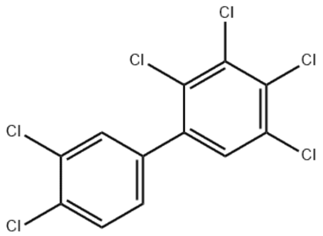

PCB 156

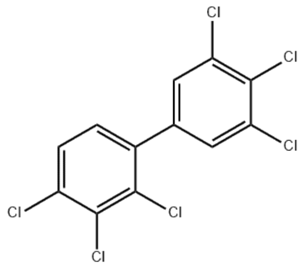

PCB 157

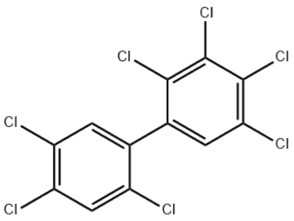

PCB 180

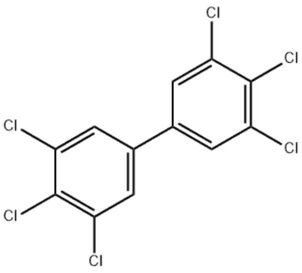

PCB 169

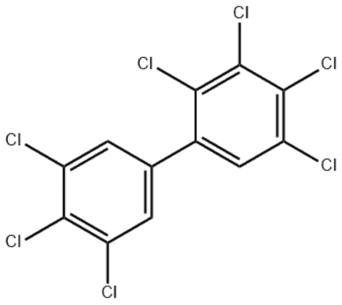

PCB 189

Figure S1. Structural formula of the selected PCB congeners.

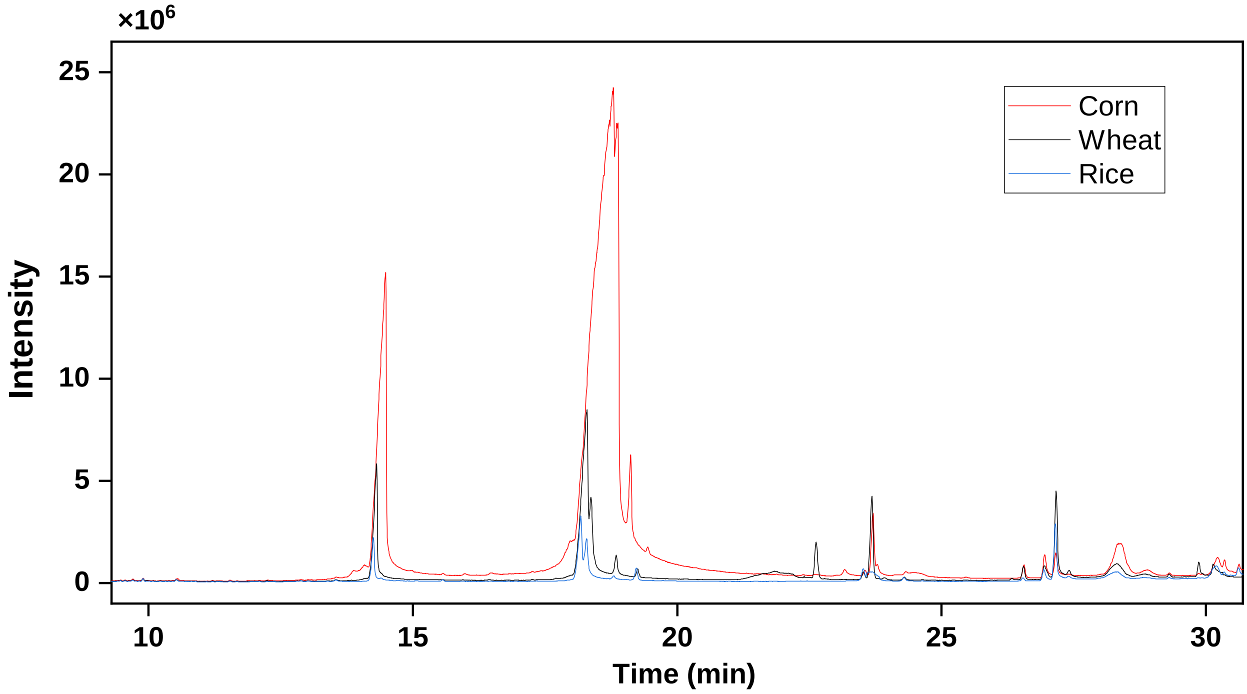

Figure S2. Total ion chromatogram of corn, wheat and rice extracts.

Table S1. Retention time, qualitative ion and quantitative ion of target PCBs.

Analyte	Group no.	Retention time (min)	Qualitative ion	Quantitative ion
PCB28	1	12.44	258,186	256
PCB52	2	13.63	220,290	292
PCB101	3	17.30	254,328	326
PCB81	4	18.51	289,294	292
PCB77	4	18.98	289,294	292
PCB123	5	20.14	328,324	326
PCB118	5	20.24	328,324	326
PCB114	5	20.78	328,324	326
PCB153	5	21.40	362,290	360
PCB105	5	21.53	328,324	326
PCB138	6	22.75	362,290	360
PCB126	6	23.20	328,324	326
PCB167	7	24.24	362,290	360
PCB156	8	25.39	362,358	360
PCB157	8	25.69	362,290	360
PCB180	9	26.32	396,323	394
PCB169	10	27.53	362,358	360
PCB189	11	29.49	396,324	394

Table S2. Linear ranges, regression equations, correlation coefficients, LODs and LOQs of 18 PCBs.

Analyte	Linear range (μg/L)	Regression equation [a]	*r*	LODs(μg/kg)			LOQs(μg/kg)			Dry matter(%)		
				Corn	Wheat	Rice	Corn	Wheat	Rice	Corn	Wheat	Rice
PCB28	0.5-50	y=210.45x-45.571	0.9986	0.04	0.05	0.04	0.1	0.2	0.1			
PCB52	0.5-50	y=111.67x-12.967	0.9985	0.08	0.1	0.08	0.3	0.3	0.3			
PCB101	0.5-50	y=140.54x-16.291	0.9983	0.08	0.09	0.08	0.3	0.3	0.3			
PCB81	0.5-50	y=190.24x-63.062	0.9983	0.08	0.08	0.08	0.3	0.3	0.3			
PCB77	0.5-50	y=194.62x-72.637	0.9985	0.08	0.08	0.08	0.3	0.3	0.3			
PCB123	0.5-50	y=169.87x-38.121	0.9983	0.07	0.08	0.07	0.2	0.3	0.2			
PCB118	0.5-50	y=184.58x-36.303	0.9985	0.07	0.07	0.07	0.2	0.2	0.2			
PCB114	0.5-50	y=170.79x-30.792	0.9983	0.07	0.07	0.07	0.2	0.2	0.2			
PCB153	0.5-50	y=126.87x-12.276	0.9982	0.09	0.09	0.09	0.3	0.3	0.3			
PCB105	0.5-50	y=172.18x-43.167	0.9984	0.08	0.08	0.08	0.3	0.3	0.3	85.9	86.8	85.7
PCB138	0.5-50	y=114.57x-12.376	0.9982	0.1	0.09	0.1	0.4	0.3	0.3			
PCB126	0.5-50	y=159.10x-43.487	0.9982	0.08	0.09	0.08	0.3	0.3	0.3			
PCB167	0.5-50	y=145.28x-26.777	0.9982	0.09	0.09	0.09	0.3	0.3	0.3			
PCB156	0.5-50	y=140.07x-25.584	0.9980	0.1	0.1	0.1	0.3	0.3	0.3			
PCB157	0.5-50	y=139.20x-22.427	0.9979	0.1	0.1	0.1	0.3	0.4	0.3			
PCB180	0.5-50	y=91.999x-5.5179	0.9978	0.1	0.1	0.1	0.4	0.3	0.3			
PCB169	0.5-50	y=137.72x-36.668	0.9980	0.09	0.1	0.1	0.3	0.4	0.3			
PCB189	0.5-50	y=113.04x-12.089	0.9980	0.1	0.08	0.1	0.3	0.3	0.3			

[a] x = concentration (μg/L), y = peak area.

Table S3. The recoveries and RSDs of proposed method for the spiked samples at different levels.

Analyte	Mean recoveries and RSDs (%, *n*=6)								
	Corn			Wheat			Rice		
	0.5 (μg/kg)	5 (μg/kg)	10 (μg/kg)	0.5 (μg/kg)	5 (μg/kg)	10 (μg/kg)	0.5 (μg/kg)	5 (μg/kg)	10 (μg/kg)
PCB28	87.8±7.8	93.5±4.8	101.5±2.7	86.4±9.4	96.5±5.0	100.2±3.5	82.5±1.7	98.1±4.1	94.3±5.2
PCB52	103.7±6.4	101.6±6.0	109.1±8.2	84.2±3.7	99.1±7.5	100.7±3.3	85.4±4.5	101.9±4.0	97.7±6.4
PCB101	96.1±6.5	96.1±4.3	108.0±7.6	86.6±5.2	98.6±5.5	102.3±4.0	79.2±4.5	99.3±3.6	99.0±5.5
PCB81	95.7±9.7	91.7±8.2	101.7±5.3	93.9±5.2	94.9±4.1	98.9±2.4	87.9±5.3	95.2±2.4	91.6±8.0
PCB77	100.2±5.1	92.3±4.5	95.8±5.3	84.1±9.1	97.8±8.1	97.4±7.1	87.0±7.3	96.0±4.1	88.1±1.5
PCB123	107.4±5.5	98.0±5.3	104.7±6.3	89.9±9.4	102.1±3.4	103.7±5.4	85.5±3.6	99.4±3.8	110.5±1.5
PCB118	104.3±3.3	93.4±5.8	105.1±3.5	84.9±4.5	101.7±6.0	102.2±4.4	85.2±6.5	99.9±2.4	96.2±3.2
PCB114	102.6±4.5	94.9±5.1	103.2±2.7	89.0±6.0	101.4±5.5	100.6±3.5	86.8±4.2	99.9±5.8	98.1±8.6
PCB153	85.3±6.4	100.3±6.0	102.1±3.1	84.5±7.0	100.2±5.9	104.1±1.9	82.7±6.0	99.8±4.7	97.8±5.2
PCB105	84.6±10.3	93.6±3.8	100.2±2.7	83.7±6.0	98.4±5.2	99.3±3.3	84.1±5.4	98.3±3.5	93.9±4.2
PCB138	97.7±8.9	97.8±2.5	94.7±3.0	84.6±7.1	96.4±4.6	106.9±4.0	86.9±7.1	95.8±2.1	97.4±2.8
PCB126	106.5±8.5	94.7±3.0	99.5±2.5	82.9±7.1	99.3±4.7	96.6±4.1	82.8±7.5	95.9±4.6	94.7±2.0
PCB167	105.9±7.1	96.7±3.0	102.7±4.5	82.2±7.5	101.4±5.0	101.9±4.0	85.2±4.9	99.3±3.6	96.3±1.9
PCB156	105.2±7.6	94.1±2.8	99.4±2.5	89.3±5.6	100.0±4.2	102.6±2.2	88.5±4.9	98.7±3.8	96.1±3.2
PCB157	99.1±8.8	91.9±3.5	98.8±3.1	79.4±3.5	98.5±4.3	101.0±4.6	82.5±5.3	99.9±5.4	96.9±3.2
PCB180	98.5±9.7	96.7±5.4	103.5±5.5	83.2±2.7	95.9±4.2	105.4±2.0	92.4±7.4	100±5.0	100.4±2.2
PCB169	87.5±5.5	95.9±5.2	98.1±1.6	79.9±6.0	99.0±4.3	98.3±2.4	89.6±4.3	98.9±3.9	95.3±5.0
PCB189	93.0±9.0	95.6±2.9	98.2±2.3	80.1±8.5	100.2±5.9	105.5±4.0	88.1±2.0	98.4±3.4	98.0±3.7
